# Structure of the Sac3 RNA-binding M-region in the *Saccharomyces cerevisiae* TREX-2 complex

**DOI:** 10.1093/nar/gkx158

**Published:** 2017-03-15

**Authors:** James M. B. Gordon, Shintaro Aibara, Murray Stewart

**Affiliations:** MRC Laboratory of Molecular Biology, Francis Crick Avenue, Cambridge Biomedical Campus, Cambridge CB2 0QH, UK

## Abstract

Transcription-export complex 2 (TREX-2, or THSC) facilitates localization of actively transcribing genes such as *GAL1* to the nuclear periphery, contributes to the generation of export-competent mRNPs and influences gene expression through interactions with Mediator. TREX-2 is based on a Sac3 scaffold to which Thp1, Sem1, Cdc31 and Sus1 bind and consists of three modules: the N-region (Sac3^∼1-100^), which binds mRNA export factor Mex67:Mtr2; the M-region, in which Thp1 and Sem1 bind to Sac3^∼100-550^; and the CID region in which Cdc31 and two Sus1 chains bind to Sac3^∼720-805^. Although the M-region of Sac3 was originally thought to encompass residues ∼250-550, we report here the 2.3Å resolution crystal structure of a complex containing Sac3 residues 60–550 that indicates that the TPR-like repeats of the M-region extend to residue 137 and that residues 90–125 form a novel loop that links Sac3 to Thp1. These new structural elements are important for growth and mRNA export *in vivo*. Although deleting Sac3 residues 1–90 produced a wild-type phenotype, deletion of the loop as well generated growth defects at 37°C, whereas the deletion of residues 1–250 impaired mRNA export and also generated longer lag times when glucose or raffinose was replaced by galactose as the carbon source.

## INTRODUCTION

The nuclear export of mature mRNAs through nuclear pore complexes (NPCs) is the culmination of the nuclear phase of the gene expression pathway. However, although the structure of NPCs has been established (1) and the Ran-based nuclear export and import of proteins by karyopherins is understood in considerable detail (2–4), mRNA export is more complex because of the necessity to first complete nuclear processing steps (such as splicing, polyadenylation, capping, etc.) and the Mex67:Mtr2 export factor is employed rather than a karyopherin (5,6). The generation of export-competent mRNPs is therefore a critical step in the gene expression pathway. Moreover, many of the nuclear steps of the gene expression pathway are coordinated, often near nuclear pores using the TREX-2 complex (6,7).

**Figure 1.**
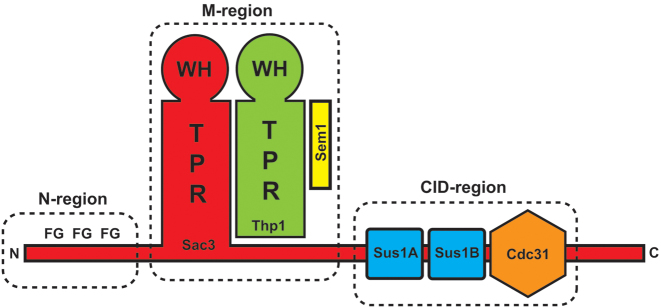
Schematic representation of the TREX-2 complex that is formed around a Sac3 scaffold (red). The TREX-2 complex can be broadly subdivided into three regions. The most N-terminal region of Sac3 (N-region) and its homologues (such as GANP from *Homo sapiens*) contain FG-nucleoporin-like motifs (7). The middle region (M-region, also called PCI-region), in which Sac3 (red) forms a complex with Thp1 (green) and Sem1 (yellow). In this region both Sac3 and Thp1 have PCI folds based on a winged helix domain (WH) and a stack of helical TPR-like motifs (15,16). The juxtaposition of the winged helix domains together with an extended patch of positive charge forms a nucleic acid binding platform (16) and this region also interacts with the Mediator complex (15). In the CID-region, residues ∼720–805 of Sac3 interact with one Cdc31 chain (orange) and two Sus1 chains (blue) in *Saccharomyces cerevisiae* and mediates binding to NPCs (17,18).

The TREX-2 complex is conserved across eukaryotes and integrates mRNA export into the gene expression pathway and, in yeast, also mediates the location of actively-expressed genes such as *GAL1* to the nuclear envelope (8–14). In *Saccharomyces cerevisiae*, TREX-2 is based on a Sac3 scaffold to which Thp1, Sem1, Cdc31 and two copies of Sus1 bind (Figure 1) and broadly speaking, the TREX-2 complex can be subdivided into three regions (7): the N-region (Sac3 residues ∼1–100), which harbours degenerate FG-like repeats similar to those seen in many nuclear pore proteins (FG nucleoporins) and which bind to the principal mRNA nuclear export factor, Mex67:Mtr2; the M- or proteasome/CSN/eIF3 (PCI)-region, consisting of Sac3 residues ∼100–551 bound to Thp1 and Sem1, which forms a nucleic acid binding module as well as docking site for components of the Mediator complex (7,15–16); and the Cdc31-interacting domain (CID)-region, in which Sac3 residues ∼720–805 bind to Cdc31 and two Sus1 chains and which, in *S. cerevisiae*, binds to the NPC to tether the complex close to the nuclear basket to facilitate localization of genes such as *GAL1* (17,18). Both the portion of Sac3 in the M-region and Thp1 have similar structures based on a PCI fold (19) in which a series of α-helical tetratricopeptide (TPR)-like repeats are followed by a winged helix motif. The juxtaposition of the winged helix motifs of Sac3 and Thp1, together with a surface patch of positive charge, contributes to the formation of a RNA-binding region in the TREX-2 complex (15,16).

The TREX-2 complex has been implicated in a spectrum of biological roles including preventing genome instability, transcription initiation, gene-gating and mRNA export (15,16,20–22). Previous work using homologues from the thermophile *Chaetomium thermophilum* indicated that the multiple FG-like repeats present on the Sac3 N-terminal region may serve as a docking site for the principal mRNA export factor Mex67:Mtr2 as well as making intra-molecular interactions with the CID-region to form an annular structure (23). However, a recent electron microscopy study of the intact *S. cerevisiae* TREX-2 complex expressed in insect cells indicated that here the CID region was orientated randomly relative to the M-region and showed no evidence to support an interaction between them mediated by the N-region (24). However, although the FG-repeats in the *S. cerevisiae* Sac3 N-terminal region are fewer and less similar to nucleoporin FG repeats, deletion of residues 1–140 generates growth and mRNA export defects (23).

Both cryo-EM and crystals obtained from the complete *S. cerevisiae* TREX-2 complex also indicated that the TPR-like repeats of the Sac3 M-region could extend past residue 250 towards the N-terminus, but, although three tubes of electron density consistent with helices could be observed, the limited resolution of these studies frustrated identification of the precise residues involved. Here we describe the 2.3 Å resolution crystal structure of a complex formed by Sac3 residues 60–550 complexed with Thp1 and Sem1. The structural model obtained indicated that the Sac3 TPR-like repeats extend to residue 137 and that residues 90–125 form a novel loop that links the distal portion of the Sac3 M-region to the TPR-like repeats in the N-terminal region of Thp1. Both the additional Sac3 TPR-like region and the novel loop linking Sac3 to Thp1 are important functionally. Deletion of the loop impairs growth at 37°C and also shows slight impairment of mRNA nuclear export, whereas deletion of the additional TPR-like region (residues 125–250) shows a pronounced mRNA export defect together with an increased lag phase when galactose is exchanged for glucose or raffinose as the carbon source. Overall this work has identified two new structural features of the Sac3 M-region that contribute to the function of the TREX-2 complex in yeast.

## MATERIALS AND METHODS

### Protein expression and purification

Sac3 (residues 60–550) cDNA was cloned into a modified TEV protease-cleavable version of pGEX-4T-1 (GE Healthcare) and co-expressed together with Thp1 and Sem1 cloned into the first and second MCS (multiple cloning site) of RSFDuet-1 (Novagen) in BL21-CodonPlus (DE3)-RIL cells (Stratagene) by isopropyl-β-D-thiogalactoside (IPTG) induction at 20°C as described (16). The Sac3^60-550^:Thp1:Sem1 complex was purified from the soluble fraction by sequential chromatography on Glutathione Sepharose 4B and Superdex 200 columns (GE Healthcare).

### X-ray crystallography

Crystals of the Sac3^60-550^:Thp1:Sem1 complex were obtained by sitting drop vapour diffusion using 200 nl of 20% PEG 3350, 0.2 M Mg formate, 100 mM MES 6.5, mixed with 200 nl of the *S. cerevisiae* Sac3^60-550^:Thp1:Sem1 complex at 16.5 mg/ml. Crystals grew over a period of 2 weeks and were harvested and then vitrified by plunging into liquid nitrogen. X-ray diffraction data were collected on beamline I03 at the Diamond Light Source (Didcot, UK). Reflections were indexed and integrated using *XDS* (25) and then scaled and merged in *AIMLESS* (26,27). An initial structural model was obtained by molecular replacement using *Phaser* in *PHENIX* (28) and the structure of the *S. cerevisiae* Sac3^255-556^:Thp1:Sem1 complex (ref. 16; PDB accession code: 3T5V) as a model. Iterative cycles of rebuilding using *COOT* (29) and refinement using *PHENIX* (28) were used to generate the final model that contained 8011 non-hydrogen protein atoms and 546 waters and which had excellent geometry (Table 1).

**Table 1.** Crystallography statistics

**Data collection statistics**	
Wavelength (Å)	0.9686
Space group	*P*2_1_2_1_2_1_
Unit cell parameters: a, b, c (Å); α, β, γ (°)	78.37, 86.63, 168.34; 90, 90, 90
Resolution range (outer shell in brackets; Å)	19.95–2.30 (2.37–2.30)
Unique reflections	51 644 (4396)
Total observations	319 802 (27 953)
<I / σ(I)>: all (outer shell)	7.5 (1.9)
R_p.i.m._: all (outer shell)	0.106 (0.979)
Completeness: all (outer shell) (%)	99.9 (100)
Multiplicity	6.1 (5.4)
Wilson B-factor	33.9
**Refinement statistics**	
Protein non-hydrogen atoms	8011
Waters	546
Bond length deviation from ideal values (Å)	0.0033
Bond angle deviation from ideal values (°)	0.53
Ramachandran favoured/outliers (%)	98.24/0
All-atom clashscore	1.37
Rwork/Rfree (%)	17.8/21.7
MolProbity (32) score (percentile)	0.87 (100)

### *In vivo* growth assays

Cells were freshly plated overnight on synthetic complete medium lacking tryptophan (SDC-TRP) plates. On the following day, they were diluted to OD_600_ = 1 after which they were spotted in 10-fold dilutions on SDC-TRP and grown for 3 days at 37°C. Growth kinetics using galactose as a carbon source for cells initially grown in glucose or raffinose were analysed by measuring OD_600_ at 6 min intervals in 96-well microplates at 30°C using a TECAN Spark 10M microplate reader and are reported as means ± SD for six determinations. Statistical significance was assessed using Student's *t*-test. Growth rates and lag times were determined as described (30). Expression levels of the mutants relative to an *ACT1* control were indistinguishable from those of *SAC3* when assessed by RT-qPCR using the SCRIPT one-step RT-qPCR greenmaster system (Jena Biosciences) following the manufacturer's protocol.

### Fluorescence *in situ* hybridization

For *in situ* hybridization, cells were grown at 30°C in selective media before being fixed. Analysis of polyA^+^ RNA export by *in situ* hybridization was carried out using Cy3-labelled 30nt oligo(dT) probes essentially as described in Amberg *et al*. (31). Cells were examined at 100X using a Nikon TE2000 microscope. Fractions of cells showing nuclear accumulation of polyA^+^ RNA were calculated by examining at least 300 cells from at least three images and are presented as mean ± SD.

## RESULTS

### Structure of the *S. cerevisiae* TREX complex M-domain

**Figure 2.**
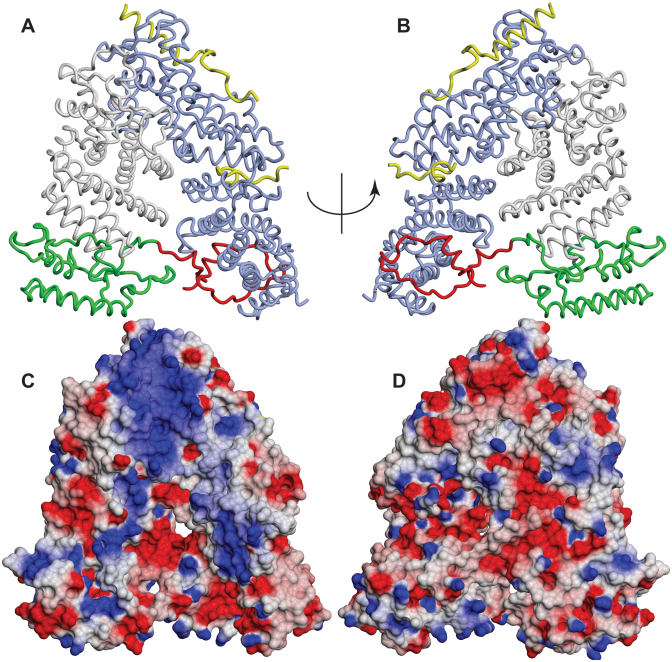
Crystal structure of the Sac3^60-550^:Thp1:Sem1 complex. (**A** and **B**) In addition to the structures of Sac3 residues 255–551 (grey), Thp1 (blue) and Sem1 (yellow) determined previously (15,16), the crystal structure of the Sac3^60-556^:Thp1:Sem1 complex showed an extension of the Sac3 TPR repeat region (green) containing three additional helices, together with a novel extended loop (red) that linked the base of the Sac3 TPR region (green) to the N-terminal region of Thp1. (**C** and **D**) Charge distribution on the surface of the Sac3^60-556^:Thp1:Sem1 complex (blue is positive, red is negative charge). There is an extensive region of positive charge stretching from the winged-helix region of both Sac3 and Thp1 (towards the top of the structure) down the distal region of Sac3 together with a distinct positively-charged patch towards the base of Thp1. These positively-charged areas are on the opposite side of the complex to the linker containing Sac3 residues 90–125.

Crystals were obtained of the Sac3^60-550^:Thp1:Sem1 complex that had *P2_1_2_1_2_1_* symmetry with *a* = 78.2 Å, *b* = 84.1 Å, *c* = 165.5 Å and which diffracted to 2.3 Å resolution using synchrotron radiation (Table 1). Phases were obtained using molecular replacement with the crystal structure of the Sac3^255-556^:Thp1:Sem1 complex (PDB accession code: 3T5V) and the electron density maps obtained after refinement and rebuilding showed residues 82–547 of Sac3, together with the Thp1 and Sem1 chains observed previously (Figure 2 and Supplementary Movie 1). The final model had an R-factor of 17.8% (Rfree = 21.7%) and a MolProbity (32) score of 0.87 (100th percentile) indicative of excellent geometry (Table 1). Except for some less-well defined regions also present in previous work, such as residues 1–26 and 42–56 of Sem1 and several loops within Sac3 and Thp1 (15,16), the chains of all three proteins could be traced reliably. In the regions that they shared, there were only minor structural differences between the present structure and the structures obtained previously for Sac3^255-556^:Thp1:Sem1 (PDB accession code: 3T5V) and Sac3^222-572^:Thp1^170-455^:Sem1 (PDB accession code: 4TRQ).

**Figure 3.**
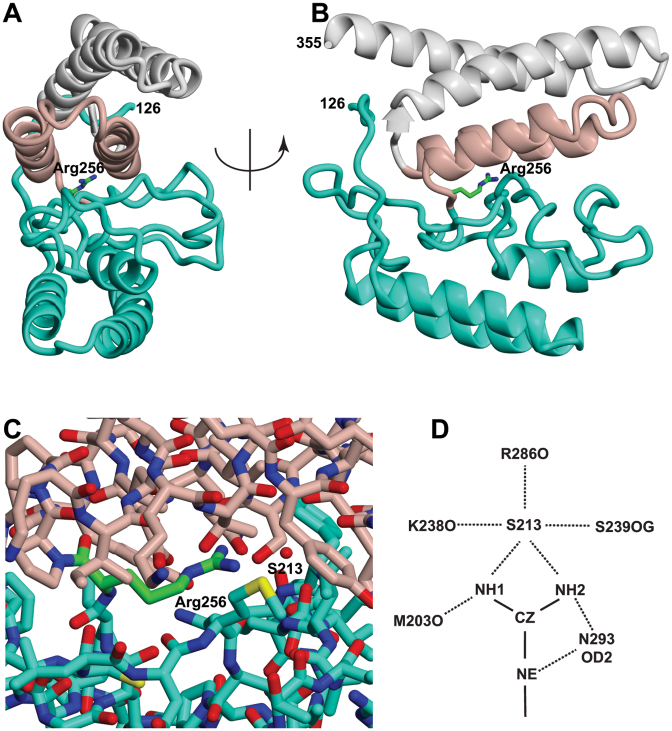
Arg256 is buried in the interface between the Sac3 TPR helices and the three additional helices. (**A** and **B**) Illustration of how Sac3 Arg256 is sandwiched between the two helices of the TPR repeat that contains residues 255–269 and 280–296 (pink) and the additional module (residues 126–250, green). Arg256 and the Sac3 255–269 and 280–296 helices formed lattice contacts in previous crystal structures and were proposed to form an interaction interface (15), but in the more complete structure of the Sac3^60-556^:Thp1:Sem1 complex they are buried and inaccessible. (**C**) Detail of the residues surrounding Arg256 and water S213 that are buried in the interface between the 255–296 TPR-like repeat and residues 125–250. (**D**) Hydrogen bond network linking Arg256 to Met203, Lys238, Arg286, Ser289, Asn293 and water S213. See also Supplementary Movies 2 and 3.

In addition to the previously determined structures of Thp1, Sem1 and Sac3 residues 255–551, Sac3 residues 82–255 were clear and easily traced, but reliable density corresponding to residues 60–81 was not observed, possibly indicating that this region was disordered. Although residues 191, 192 and 244 were in poorly-defined loops, the connectivity and path of the main chain was clear and showed three roughly parallel α-helices together with a novel elongated loop that linked this region of Sac3 to Thp1. Electron microscopy (24) had shown three clear cylinders of additional density, consistent with α-helices, past residue 253 at which the original structural models (PDB accession codes: 3T5V and 4TRQ) were truncated and had suggested that the Sac3 TPR-like repeat region extended further towards the N-terminus. The Sac3^60-550^:Thp1:Sem1 crystal structure indicated that the TPR-like repeats of Sac3 indeed extend to residue 137 and identified unequivocally the residues in the three major helices. Sequence analysis using the PSIPRED package (http://bioinf.cs.ucl.ac.uk/psipred—ref. 33) had indicated that residues 143–152, 154–177, 206–215 and 233–240 had a high potential for forming α-helices, whereas residues 1–142 showed little potential for forming secondary structure. However, the crystal structure showed that the residues that contributed to the three additional helices were 137–153, 157–177 and 205–213. These three additional helices both extend the TPR-like solenoid of the Sac3 M-region and also cap it. Sac3 residues 126–137 were arranged along one side of the three-helix stack and possibly contributed to its stability. Remarkably, in the Sac3^60-556^:Thp1:Sem1 complex crystals, Sac3 Arg256 was completely buried between the PCI region determined previously (residues 250–556) and the additional three helices (residues 137–250), where it formed a complex network of H-bonds to Asp293, Ser289 and Met203, and to Arg286, Ser289 and Lys238 through water S213 (Figure 3 and Supplementary Movies 2 and 3) that was augmented with hydrophobic interactions with Ile256, Leu261 and Cys200. Arg256 and residues interacting with it in this region were conserved between different species (Supplementary Figures S1 and 2) as would be anticipated for buried core residues.

**Figure 4.**
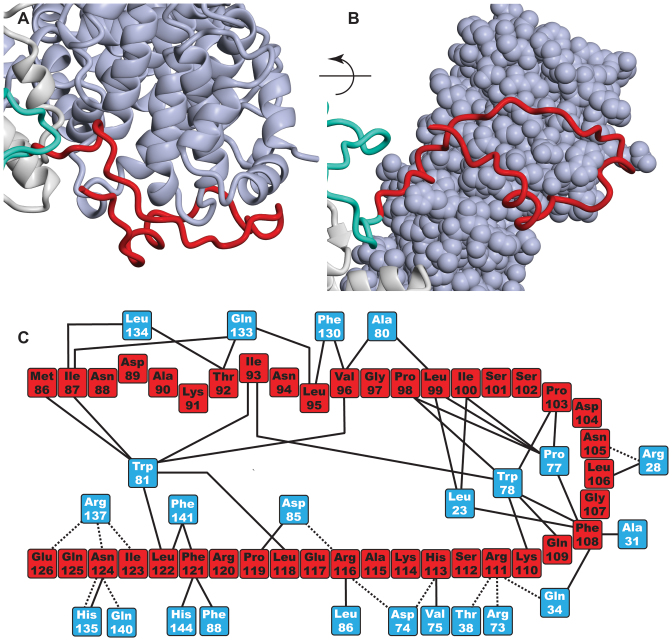
Sac3 residues 90–125 form an extended loop linking the distal end of the TPR region to Thp1. (**A** and **B**) Illustration of how the loop (red) stretches out from Sac3 (grey) to encircle the N-terminal region of the Thp1 PCI region (blue) with which it makes an extensive number of contacts and buries 1528 Å^2^ of surface area. Except for a small helix (residues 89–95), the residues in the loop lack regular secondary structure. (**C**) Schematic illustration of the interactions between residues in the Sac3 extended loop (red) and Thp1 (blue).

In addition to the three additional helices of the TPR-like repeat region, the crystal structure of the Sac3^60-556^:Thp1:Sem1 complex showed that Sac3 residues 90–125 formed a remarkable extended loop that linked the distal region of the Sac3 TPR-like region to the N-terminal region of Thp1 (Figures 2A, B, 4 and Supplementary Movie 1). With the exception of two short helices (residues 90–96 and 103–106), this loop did not contain extensive secondary structure and was generally in an extended conformation. The loop made extensive contacts with Thp1 (Figure 4) and buried 1528 Å^2^ of surface area. Although the interface was dominated by hydrophobic side chains, notably Thp1 Pro77, Trp78 and Trp81 on Thp1 and Ile87, Ile93, Pro98, Leu99, Ile100, Phe108 and Phe123 on Sac3, 12 putative H-bonds also contributed to the interaction.

Previous studies identified a large surface patch of positive charge located over the winged helix regions of both Thp1 and Sac3 that extended somewhat towards the distal region of the Sac3 PCI fold and which has been implicated in interactions with nucleic acids (16) and the Mediator complex (15). This patch continued over the additional helices (Figure 2C and D) and so is more extensive than thought. However, the second positively charged cluster centered on Sac3 Arg256 that has been proposed to be involved in the interaction with Mediator (15) was buried by the residues in the additional TPR region below residue 250 and so was inaccessible (Figure 3) in the complete M-region structure. Generally the regions of positive surface charge were located on the opposite side of the complex to the loop formed by Sac3 residues 90–125 (Figure 2A and B) indicating that this loop may not interact directly with nucleic acids.

In summary, the crystal structure of the Sac3^60-550^:Thp1:Sem1 complex identified two new structural features in the Sac3 M-region. In one, residues 137–250-fold into three additional helices that continue the stack of TPR-like helices previously identified in residues 255–550 and which appeared to be stabilized by residues 126–136 that lie along one side of these helices. In the second, residues 90–125 formed a remarkable loop that reaches out from Sac3 to connect it to the N-terminal region of Thp1.

### Functional roles of the new Sac3 elements

Sac3 deletion mutants were used to explore the functional role of the extended loop (residues 90–125) and the continuation of the TPR region (residues 125–250) to function in terms of growth rate, *GAL* induction, and mRNA export.

**Figure 5.**
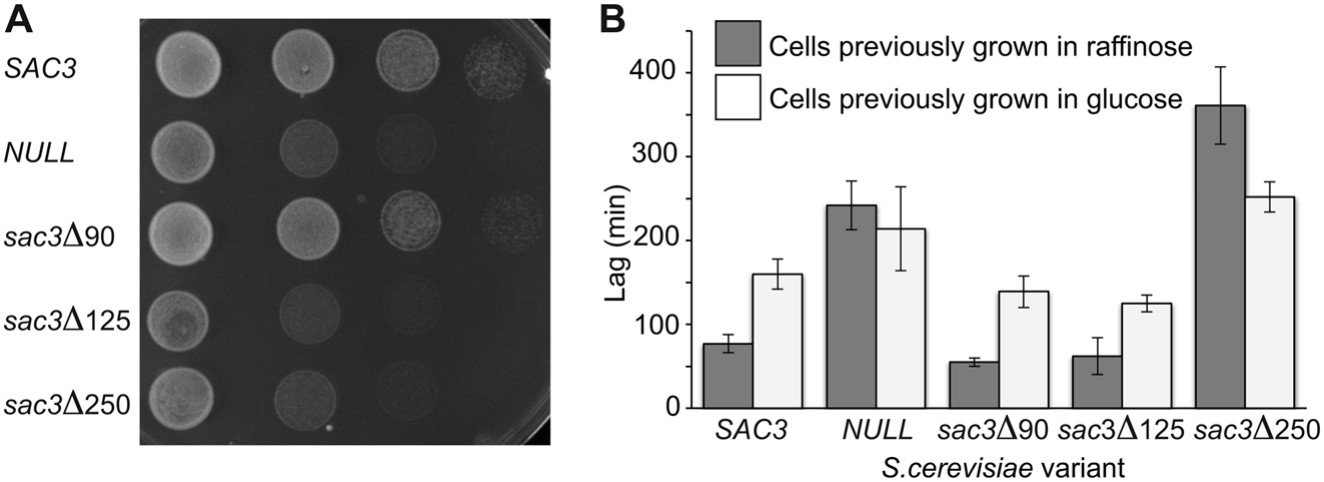
Influence of different Sac3 regions on growth. (**A**) Serial dilutions of *sac3 NULL* cells carrying plasmid-borne *SAC3* or different *SAC3* truncations. Cells were grown at 37°C for 3 days. Although deletion of resides 1–90 (*sac3Δ90*) grew similarly to *SAC3* cells, deletion of the extended loop (*sac3Δ125)* produced a growth defect similar to *sac3 NULL* cells. (**B**) Lag times for *Saccharomyces cerevisiae* cells transferred from either glucose or raffinose to galactose medium. Cells previously grown in glucose showed longer lag times than cells previously grown in raffinose. *SAC3, sac3Δ90* and *sac3Δ125* showed similar lag phases, whereas *sac3 NULL* cells and *sac3Δ250* cells showed longer lag times (*P* < 0.05). Bars represent SDs from six determinations.

Deletion of the Sac3 extended loop (*sac3Δ125*) impaired cell growth at 37°C to levels comparable to deletion of *SAC3* (Figure 5A). Although deletion of *SAC3* generates a growth defect at 37°C (10,13,34), previous work had shown that deletion of residues 1–60 did not impair growth at 37°C, whereas deletion of residues 1–140 (23), the R256D mutation (15) or mutation of positively-charged residues in the winged-helix domains of either Sac3 or Thp1 (16), generated severe growth defects at 37°C. We found that, whereas deletion of residues 1–90 (*sac*3*Δ90*) had a negligible impact on growth at 37°C, deletion of these residues together with the extended loop (*sac3Δ125*) showed a considerable growth defect that was comparable to that seen in *sac3 NULL* cells (Figure 5A).

Although the extended loop (residues 90–125) did not appear to influence the lag time when galactose was substituted for glucose or raffinose as the carbon source, deletion of both the loop and the TPR extension (*sac3Δ250*) resulted in a substantial increase in the lag time (Figure 5B). The lag time for cells initially grown in glucose was longer than for cells grown in raffinose because, although in both cases it is necessary to induce the *GAL* genes needed for galactose metabolism, it is also necessary to remove the repression of the *GAL* genes present when glucose is the carbon source. Budding yeast grow best using glucose as a carbon source, but can use galactose if glucose is absent. In the presence of glucose, the genes of the galactose (*GAL*) network are repressed by several different mechanisms (including recruitment of the Mig1 DNA-binding repressor and the Cyc8:Tup1 co-repressor complex) acting in concert (35,36). However, this repression is removed when an alternative carbon source, such as galactose, raffinose or glycerol, is substituted for glucose and is facilitated by desumoylation of Cyc8 and Tup1 by Ulp1 that is located at NPCs (37). Although in glucose medium the *GAL7-10-1* gene cluster is distributed throughout the nucleus, this cluster becomes localized to the nuclear envelope in either raffinose or galactose medium (13), bringing it into close proximity to Ulp1 facilitating removal of the Mig1-based repression (37). However, in the presence of raffinose or glycerol, Gal80 still remains bound to the Gal4 activator and represses its activity. In the presence of galactose, Gal80 is released from Gal4, activating the *GAL* genes. Consequently, the lag phase when cells are transferred directly from glucose to galactose medium is reduced when cells are initially grown in raffinose before being transferred to galactose. We found that both *sac3 NULL* and *sac3Δ250* cells showed significantly (*P* < 0.05) longer lag times when raffinose was replaced by galactose as the carbon source, whereas *sac3Δ90* and *sac3Δ125* cells showed lag times of the order of 50 min, similar to that observed for *SAC3* cells. The lag phase with *sac3Δ250* cells was considerably longer (*P* < 0.05) even than that seen with *sac3 NULL* cells (Figure 5B) perhaps as a result of its sequestering other cellular components as seen with other Sac3 mutants (34). A similar pattern was seen in cells transferred directly from glucose to galactose medium, although in this case the lag times were generally longer (Figure 5B). Again only *sac3 NULL* and *sac3Δ250* cells showed lag times significantly (*P* < 0.05) longer than wild-type cells. Taken together, these results indicate a role for Sac3 residues 126–250 (that correspond to the extension of the Sac3 TPR domain) in the expression of the *GAL* genes.

**Figure 6.**
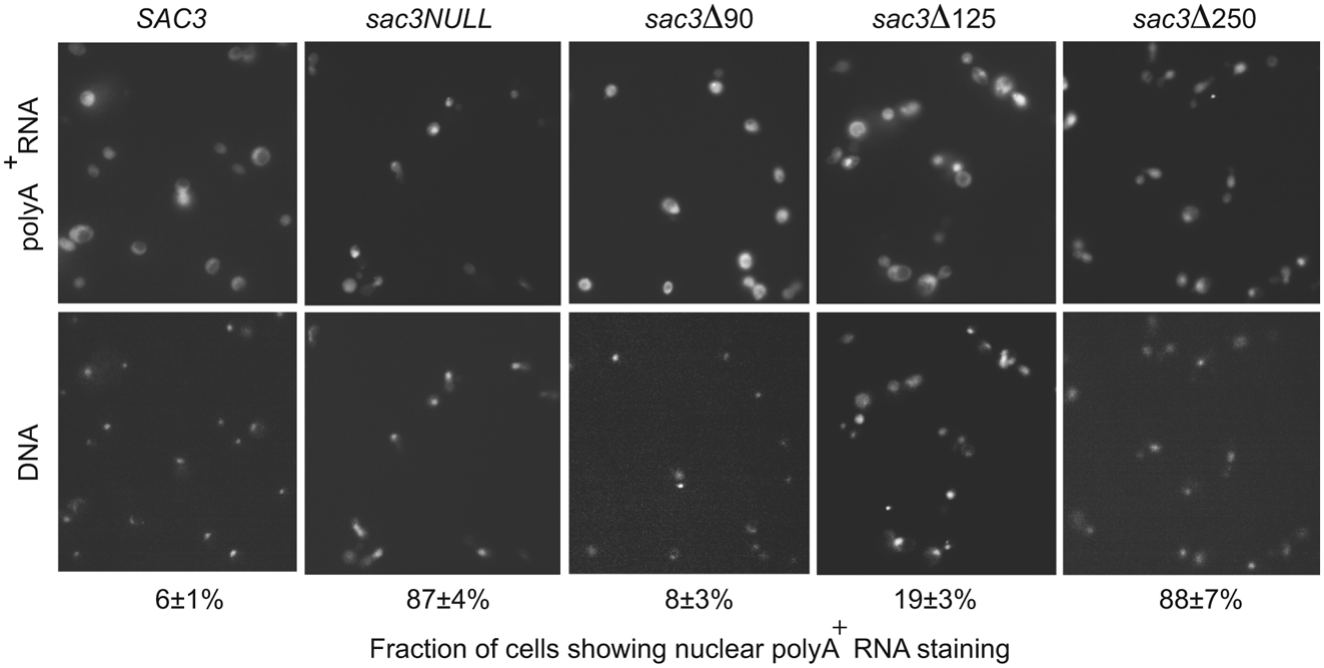
Contributions of different regions of Sac3 to mRNA nuclear export. Levels of nuclear accumulation of polyA^+^ RNA with different Sac3 variants were assessed using FISH with Cy3-labelled 30 nt oligo-(dT) probes. *Sac3Δ90* showed 8% of cells with increased nuclear staining, which was similar to *SAC3* cells (6%); *sac3Δ125* cells showed a slight increase in nuclear staining (19%), whereas most *sac3Δ250* cells (88%) showed nuclear accumulation of polyA^+^ RNA similar to that seen with *sac3 NULL* cells (86%), indicting that the nuclear export of mRNA had been impaired by this deletion.

In addition to increasing the lag time when galactose was substituted for glucose or raffinose as a carbon source, the *sac3Δ250* variant showed polyA^+^ RNA export defects, with 88% of cells showing a level of nuclear retention of polyA^+^ RNA when examined by FISH (Figure 6). The *sac3Δ125* variant showed a slight impairment, with 19% of cells showing some nuclear accumulation in FISH, which, although statistically significant (*P* < 0.05), was marginal when compared to the defect seen with *sac3 NULL* or *sac3Δ250* cells. The *sac3Δ90* variant showed only 8% of cells with nuclear accumulation of polyA^+^ RNA and was similar to wild-type cells that showed 6% (Figure 6).

**Figure 7.**
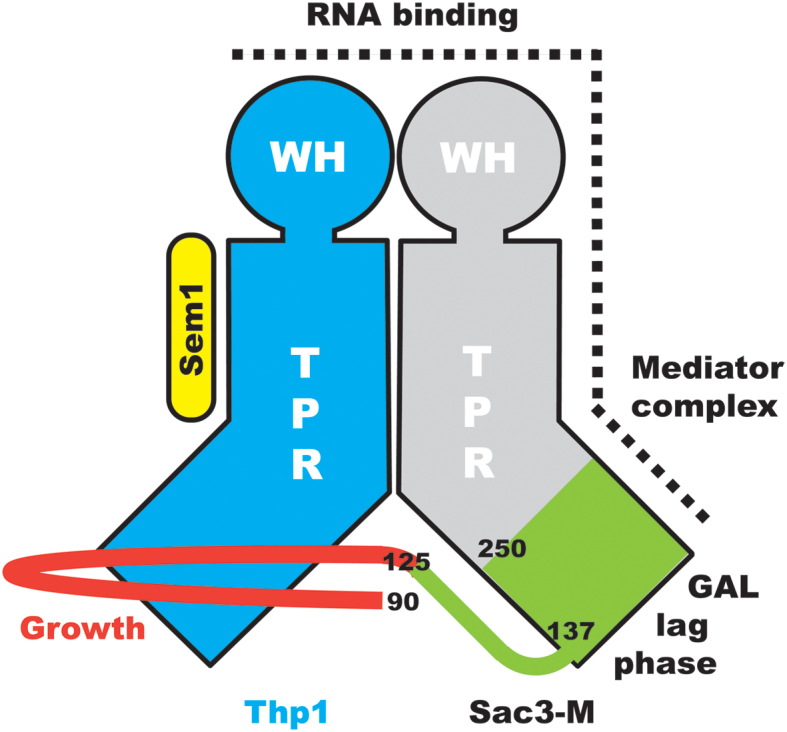
Schematic illustration of the structure of the TREX-2 M-region. Both Sac3 and Thp1 have PCI folds based on a series of α-helical TPR repeats together with a winged helix (WH) domain, whereas Sem1 lies along one side of Thp1. The structure of the Sac3^60-556^:Thp1:Sem1 complex has identified two new structural features in Sac3: an extension of the TPR region from residue 250 to residue 127 (green) and an extended loop comprising residues 90–125 that links the distal portion of the Sac3 TPR region with Thp1. A cluster of positively-charged residues that extends from the Thp1 winged helix domain and down one side of Sac3 appears to be important for binding RNA. The extended loop (residues 90–125) is important for growth, whereas residues 126–250 are important for the induction of *GAL* genes when cells are moved to galactose as a carbon source. Other studies (15) have indicated that positive residues in the Sac3 TPR region also function in conjunction with the mediator complex.

In summary, the data obtained with the deletion mutants indicate that, although the loop containing residues 90–125 is important for growth, its deletion does not impair mRNA export greatly nor the lag times seen when galactose is substituted as the carbon source, whereas the TPR extension is important for both of these functions.

## DISCUSSION

Although previous studies proposed that Sac3 residues 220–250 are inherently disordered (15), results obtained by cryo-EM and crystallography pointed towards there being considerable secondary and tertiary structure in this region and indicated that there are three helices present in the Sac3 chain before residue 255 (24). Moreover, even when they are not in secondary structure elements, the positions of residues 90–250 were well defined and clearly not disordered. The 2.3 Å resolution crystal structure of the Sac3^60-556^:Thp1:Sem1 complex demonstrated that the TPR-like repeats of the PCI fold of the Sac3 M-region indeed extend past residue 255 to residue 137, and also showed the presence of a novel loop (residues 90–125) that links the Sac3 TPR region to Thp1 (Figures 2 and 7 and Supplementary Movie 1). The sequences of Sac3 homologues from both yeast (Supplementary Figure S1) and vertebrates (Supplementary Figure S2) were conserved between residues 90 and 250, consistent with both the helices and the extended loop being retained.

The Sac3^M^:Thp1:Sem1 region forms an interaction platform by which the TREX-2 complex interacts with both mRNAs and the Mediator complex (15,16) and so the demonstration that the TPR-repeat region extends substantially below residue 255 indicates that the interaction interface between TREX-2 and these components may be more extensive than previously thought. Moreover, the new structural information indicated that results obtained previously with some Sac3 truncation constructs should be interpreted cautiously because the truncations were sometimes located within secondary structural elements and so could have disrupted the conformation locally. Thus, for example, although deletion of Sac3 residues 1–140 generates both growth and mRNA export defects that are similar to those observed in a *SAC3 null* strain (23), residues 137–140 are contained within a helix and residues 126–136 appear to contribute to the stability of the distal TPR repeat. Moreover, this construct lacked the 90–125 loop and so, because the Sac3*Δ125* mutant showed a similar growth defect (Figure 5B), the growth defect observed could derive from this source. Similarly, although deletion of residues 1–221 generated crystals in which Sac3 residues 222–252 were proposed to be disordered (15), this disorder could also have resulted from deletion of the three additional helices (residues 137–153, 157–177 and 205–213). Residues 222–252 were not disordered when the longer Sac3^60-550^ construct was employed (Figure 2A, B and Supplementary Movie 1). It has also been proposed (15) that because the Sac3 helices encompassing residues 258–269 and 280–296 contributed to crystal packing contacts in two different crystal forms, they could represent a physiologically relevant interaction site. However, this interface is buried by the extension of the TPR domain in the Sac3^60-550^:Thp1:Sem1 crystals (Figure 3 and Supplementary Movies 2 and 3) and so is inaccessible in the complete M-domain. It is, however, likely that in constructs in which residues 1–250 were absent, this exposed interface could have been somewhat sticky and could have contributed to crystal packing contacts as a result.

The TREX-2 complex functions in a number of different contexts (including mRNA export, integrating nuclear steps of the gene expression pathway, transcription of genes such as *GAL*) and different regions of the complex appear to contribute to each. The localization of TREX-2 to NPCs, which appears to be important for all of these functions, appears to derive primarily from the interaction of the CID domain (Sac3 residues 720–805, together with Cdc31 and two Sus1 chains) with Nup1, a component of the NPC nuclear basket (10,17,18), whereas the M- and N- regions appear to be important growth, mRNA export, localization of genes such as *GAL 7-10-1* cluster and the integration of many of the nuclear steps in the gene expression pathway. Previous work has implicated positively charged residues in the Sac3 M-region with the interaction with both RNAs (16,38) and with the Mediator complex (15). In the latter case, it is unlikely that one of the Sac3 residues proposed as interacting with the Mediator complex, Arg256, is involved directly because it is inaccessible (Figure 3 and Supplementary Movies 2 and 3). Mutating Arg256 to Asp would be expected to disrupt the interface between the residues in the distal helical region of the Sac3 PCI fold (residues 125–250) and those in the next TPR repeat and the local loss of structure could instead account for the this variant not interacting with the Mediator complex. Although further work will be required to define the precise role of the R256D mutant, because Sac3 residues 255–557 alone form a well-ordered structure, any disruption due to mutating Arg256 might be restricted to the distal region of the Sac3 TPR region and so account for the ability of this mutant to still bind to Thp1.

Although further work will be necessary to define the precise function of the extended loop formed by Sac3 residues 90–125, this loop is clearly important and deleting residues 1–125 (*sac3Δ125*) generated a growth defect at 37°C similar to that observed with the *sac*3 *NULL* strain (Figure 5A), whereas deletion of residues 1–90 (*sac3Δ90*) displayed wild-type growth. The considerable surface area buried by this loop's binding to Thp1 (1528 Å^2^) would be consistent with this interaction being relatively strong and it could, for example, function to retain the position of the distal region of the Sac3 M-region (residues 125–250) relative to Thp1. The lack of clusters of positive charge in this region (Figure 2C and D) suggests that it is probably not involved directly in interactions with nucleic acids and its small impact on mRNA export was probably a result of its deletion altering the structure of Sac3 residues 125–250, especially since impairment of mRNA export by TREX-2 variants does not necessary translate into any reduction in growth rate (see, e.g. 20, 24, 35). Thus, the phenotype of the *sac3ΔCID* strain shows a slightly reduced growth rate and a moderate mRNA export defect when compared with the *sac3* null strain, which is strongly impaired in mRNA export. However, deletion of residues 1–250 showed more pronounced impairment of mRNA export indicating that, in addition to the major positive patch centered on the winged helix regions of Sac3 and Thp1, residues in the distal region of Sac3 past residue 250 that encompassed the three additional helices made an important contribution to this function. Indeed, as seen in Figure 2C, the positively-charged region did extend somewhat towards the base of the Sac3 TPR domain and would be consistent with an analogous extension of the mRNA binding region.

Although the loop encompassing Sac3 residues 90–125 impaired growth at 37°C (Figure 5A), it did not alter the lag times observed at 30°C when galactose was substituted as a carbon source with cells grown initially in either glucose or raffinose (Figure 5B). *Sac3 NULL* cells showed increased lag times for cells grown initially in either glucose or raffinose and remarkably *sac3Δ250* cells showed an even greater increase, especially in cells initially grown in raffinose. Because the lag phase when raffinose is replaced by galactose requires only activation of *GAL1*, it is shorter than when glucose is replaced by galactose because then both derepression and activation are necessary. Previous work has shown that TREX-2 facilitates the localization of *GAL* genes to NPCs when either raffinose or galactose is used as a carbon source and, in addition to derepessing/activating the *GAL* genes, this localization also appears to generate a negative feedback loop that is important when galactose is removed (13). Although the localization of TREX-2 itself to NPCs is mediated primarily by an interaction between the CID region and proteins of the nuclear basket such as Nup1 (10,17), Schneider *et al*. (15) have shown that the M- region of TREX-2 is also required for localization of the *GAL7-10-1* cluster to NPCs and the increase in lag times seen with *sac3Δ250* cells is consistent with the region containing Sac3 residues 125–250 contributing to this function.

In summary, the 2.3 Å resolution the crystal structure of the Sac3^60-550^:Thp1:Sem1 complex has demonstrated that the TPR-like repeat region of the Sac3 chain extends further towards the N-terminus than had been proposed previously and, in addition to three additional helices that extend the TPR-like domain, Sac3 residues 90–125 form an extended loop that links the distal regions of the Sac3 and Thp1 PCI domains (Figure 7). Deletion mutants indicate that the additional TPR-like region of Sac3 is important for both mRNA export and for orchestrating the expression of *GAL* genes when galactose is substituted for either glucose or raffinose as a carbon source, whereas deletion of the Sac3^90-125^ loop results in a growth defect at 37°C.

## ACCESSION CODE

Coordinates and structure factors for the Sac3^60-550^:Thp1:Sem1 complex have been deposited in the protein data bank with accession code 5UBP.

## Supplementary Material

Supplementary Data
