# Supplementary material for: Structure of the Sac3 RNA-binding M-region in the Saccharomyces cerevisiae TREX-2 complex
Source: Nucleic Acids Res. 2017 Mar 15;45(9):5577–85. doi: 10.1093/nar/gkx158 (PMC5435946; doi:10.1093/nar/gkx158)
Supplement: Supplementary Data [file gkx158_Supp.zip › nar-03734-r-2016-File009.pdf]

**SUPPLEMENTARY MATERIAL**

Structure of the Sac3 RNA-Binding M-region in the *Saccharomyces cerevisiae* TREX-2 Complex by James M. B. Gordon, Shintaro Aibara and Murray Stewart.

*MRC Laboratory of Molecular Biology, Francis Crick Avenue, Cambridge Biomedical Campus, Cambridge CB2 0QH, U.K.*

**Supplementary Movie 1: Crystal structure of the Sac3$^{60\text{-}550}$:Thp1:Sem1 complex.** Thp1 is blue, Sem1 is yellow. The structure of Sac2 residues 255-547 determined in previous studies is shown as grey. The two new features established by the present study are the extension of the TPR region (residues 126-255; green) and the long loop (residues 90-125; red) that links the distal region of the Sac3 PCR fold to Thp1. See also Figure 2.

**Supplementary Movie 2: Environment of Sac3 Arg256.** In the Sac3$^{60\text{-}550}$:Thp1:Sem1 complex Arg256 is inaccessible and is buried between the helices formed by Sac3 residues 255-269 and 280-296 (pink) and the addition to the TPR module (residues 126-250, green). Water S215 is also buried in this interface and contributes to an extensive series of H-bonds involving Arg256 (see Figure 3).

**Supplementary Movie 3: Electron density map in the region of Sac3 Arg256**. In the Sac3$^{60\text{-}550}$:Thp1:Sem1 complex Arg256 is inaccessible and is buried between the helices formed by Sac3 residues 255-269 and 280-296 (pink) and the addition to the TPR module (residues 126-250, green). Water S215 is also buried in this interface and contributes to an extensive series of H-bonds involving Arg256 (see Figure 3).

```
Scer    76 ENKPDKKKKYMINDAKTIQLVGPLI--SSPDNLGFQKRSH-K-ARELPRFLINQEPQLE   130
Sbay    80 EKAADQKKKYMINDQNTIQLIGPLI--ASPESLGFQKKPH-N-PRELPRFLINQEPQLK   134
Smik    76 GTKADQKKKYMVADEKTIQIVGPLV--ASPDILGFQKRSH-K-PRELPRFLINQDPQLE   130
Spar    76 ENKPDQKKKYMTSDDKTIQLVGPLV--SSPDSLGFQRRSH-K-PRELPRFLINQEPQLK   130
Agos    55 D-RQHRKMGSARLSGAQDDAVGAVAERFSPETMGFQRFAH-K-KRETPRYMLQQTPLLW   110
Sklu    84 I-SVQNRTLMPHATPDQDSSIGPLVQKVNPEQLGFQHYSH-P-FRDLPRYLIQQIPQLK   139
Clus   106 AVSAVVDGPSQNFTTNEVAQVGAVF--EDPSKLGFMKSQQKKKSRATPRYMLSQPRLLV   162
Cgui    80 ---HKPVRGTDGFSPSEIAETGPIV--ADPTSLGYKKQ--TRTPRPKPKYLLPQQKVLS   131
Dhan   109 VIPRQKPDMVQQYTPGEINSTGGLI--LNPEQFGFQRNHKTISPRPIPKYLLHQPRLLV   165
Cpar    94 KNPNQTSGQAQSFSADEISVTGPLF--PDPQALGFQPLKKNVTPRPIPKYFLTQPKCLD   150
Ctro    85 IRPTMSSNKIQVFDDQDVQITGPLF--LNPESLGFSNRKIQDSARPVPKYFLTQPKLLH   141
Calb    83 STTSKQISNLQVFKESDIVATGALF--EQPERLGFNHRRP-TEVRSIPKYFLTQPKLLY   139
                                * :    .*  :*:         *  *:::: *   *

Scer   131 KRAFVQDPWDKANQEKMISLEES--IDDLNELYETLKKMRNTERSIMEEKGLVDKADSA   187
Sbay   135 QRKFVQDPWDKANQQKMLTLEES--IDDLNELYETLKQMRNKERSIMEDKGLVDKADSA   191
Smik   131 KREFVQDSWDKINQQKMLSLEES--IDDLNELYETLKKMRNTERSIMEEKGLVDKADSA   187
Spar   131 KRIFVQDPWDKANQEKMISLEES--IDDLNELYETLKKMRNTERTIMEEKGLVDKADSA   187
Agos   111 PQQFAQDPWDASNQKKMLSLEGS--IADVTELWETLKKIRDVERKIMEQKGLVDRADFA   167
Sklu   140 PKQFIQDPWDKSNQEKMLLLEDS--VSDVDELYETLKKMRDVERKVMEDRGLVDKADLA   196
Clus   163 TPPFHQDPWDFENQAKMTDIEAKNNGSDYQGIYEEFQKMREVERKKMEELGLVDAENTR   223
Cgui   132 APPFVQDKWDEDNQQKMLEMESKSNGQDYQGLYEELQAMREVERKHMELAGLVDAENTR   190
Dhan   166 TPIFSQDAWDKQNQAKMLSMEEQNSGTDYQGLYEEFQRMRETERKQMETLGLVDAENIS   224
Cpar   151 TPPFVQNEWDRQNQIKMEQMESANQGKDYQGLYEDLQKLREIERKEMEELGLVDAENTA   209
Ctro   142 TPEFTPNQWDKENQEKMTIMEVQNQGKDYQGLYEDMQKLREIERSKMEELGLVDAENSA   200
Calb   140 TPEFVQNPWDQENQNKLTLMEAENGGRDYQGLYEDMQKLREIERTKMEELGLVDAENVA   198
              *  : **  ** ::   :*      *  ::*  :: :*: **  **  ****  :

                          203                                 238
Scer   188 KDLYDAIVFQGTCLDMCPTFERSRRNVEYTVYSYEKNQPNDKKASRTKALKVFARPAAA   246
Sbay   192 KDLYDAIVFQGTCQDMCPIFERSRRNVEYTVFSYEKNGPDDKKASRTRALKVFARPAAA   250
Smik   188 KDLYDAIVFQGTCLDMCPIFERSRRNVEYTVYSYEKNQPNDKKASRTKALKVFARPAAA   246
Spar   188 KDLYDAIVFQGTCLDMCPIFERSRRNVEYTVYSYEKNQPNDKKASRTRALKVFARPAAA   246
Agos   168 KDLNDAIVFQGTCQDMCPIFERARRSVENNVVRYEKENPTDKRISRFRALKVFARPAAA   226
Sklu   197 KDLNDAIVFQGTCLDMCPIFERARRSVENNVVRYEKDGSVNSRISRFRALKVFARPAAA   255
Clus   224 KDLNDAIYFQGTCLDMCPVFERTRRALENNVKTLEKD-PTTNKISRERAVKAFSRPAAG   281
Cgui   191 KDLNDAISFRGTCQDMCPVFERVRRALENNVQSLERD-PVTNKISRSRAVKAFSRPAAG   248
Dhan   225 KDLTDAISFQGSCLDMCPIFERVRRALEKNVKALEKD-PNTNKISRTRAIKAFSRPAAG   282
Cpar   210 KHLNEAIAFQGSCLDMCPVFERVRRQLENNVNVLERD-PSTNKITKEKAVKAFSRPAAG   267
Ctro   201 KDLTEAISFQGSCLDMCPVFERVRRQLENNVKALEKD-PMTNKISRERAVKAFSRPAAG   258
Calb   199 KDLTEAISFSGSCLDMCPVFERVRRQLENNVKALEKD-PISNKISRERAVKAFSRPAAG   256
           *.* :** * *:* **** *** ** :* .*   *:      .:  : :*:*.*:****.

            256                            289    293
Scer   247 AAPPLPSDVRPPHILVKTLDYIVDNLLTTLPESEGFLWDRMRSIRQDFTYQNYSGPEAV   305
Sbay   251 AAPPLPSDVRPPHILVKTLDYIVDHLLATLPESEGFLWDRMRSIRQDFTYQNYSGPEAV   309
Smik   247 AAPPLPSDVRPPHILVKTLDYIVDNLLTTLPESEGFLWDRMRSIRQDFTYQNYSGPEAV   305
Spar   247 AAPPLPSDVRPPHILVKTLDYIVDNLLTTLPESEGFLWDRMRSIRQDFTYQNYSGPEAI   305
Agos   227 AAPPLPSDVRPPHVLVKTLDYIVAHILQLLPDCESFLWDRMRSIRQDFTYQNYCGPEAI   285
Sklu   256 AAPPLPSDVRPPHILVKTLDYIVENIVPLLPDCESFLWDRMRSIRQDFTYQNYSGPEAV   314
Clus   280 QPPPMPSDVRPPHVLMKTLDYIVDNFVDQLPEAHSFIWDRTRSIRQDFIYQNFYGSEAI   338
Cgui   249 QPPPLPSEVRPPQVLKSTLDYLVDEILPQLPAAHPFIWDRTRSIRQDFTYQNYFGPEAI   307
Dhan   283 QPPPLPSEVRPPHILKQTLDYLIENIVPQLPEAHSFVWDRTRSIRQDFTYQNFFGPEAI   341
Cpar   268 QPPPLPSEVRPPHVLQTTLNYLIENVVDKLPESHSFLWDRTRSIRQDFTYQNSFGPEAV   326
Ctro   259 QPPPLPSDVRPPFVLKQTLDYLVDNILSQLPEAHSFIWDRTRSIRQDFTYQNSFGPEAI   317
Calb   257 QPPPLPSDVRPPHVLSQTLNYLVDNILQQLPEAHSFIWDRTRSIRQDFTYQNNFGPEAV   315
             **:**:**** :*  **:*:: ..:  ** .. *:*** ******* ***  ****:
```

**Figure S1. ClustalOmega alignment of Sac3 sequences between different budding yeast.** The high conservation between residues 90 and 250 indicates that the extended loop (residues 90-125) and the extension of the TPR-like region (residues 125-250) are conserved. Arg 256 (red) and the residues surrounding it , such as Met203, Lys238, Arg286 and Asp289 (cyan) are also conserved. Conservation is indicated below the sequences with * representing identity, : representing high homology and . representing lower homology. Abbreviations: Scer *S.cerevisiae*, Sbay *S.bayanus,* Smik *S.mikatae*, Agos *A.gossypii*, Sklu *S.kluyveri*, Clus C.*lusitaniae*, Cgui *C.guilliermondii*, Dhan *D.hansenii*, Cpar *C.parapsilosis*, Ctop *C.tropicalis*, Calb C.*albicans*. This alignment was obtained using the EBI server (http://www.ebi.ac.uk/Tools/msa/clustalo).

```
S.cerevisae  76 ENKPDKKKKY-MINDAKTIQLVGPLISSPDNLGFQKRSHKA--RELPRFLINQEPQLEK-      131
D.rerio     560 PARPSEIKDV--IEDPE--SASALLQSPPIHKPLPRANPISMSGSLTKGSPIKKSSIAKT      615
H.sapiens   513 PFSLKEKKPGDGEVSPS--TEDAPFQHSPLGKAAGRTGASS--L-LNKSSPVKKPSLLKA      567
G.gallus    330 EFSSKEKTASEDEGRQN--SEEQNYQHSPLRKPLIRSSASA--VMPGRSSPAKKPGLRKA      385
                    .: .        .           *      : .  :      :    ::  : *

S.cerevisae 132 RAF-VQDPWDKANQEKMIS--LEESIDDLNELYETLKKMRNTERSIMEEK--GLVDKADS      186
D.rerio     616 LQFESEGPFESVSEERSIERPINILPSVLQPLVGQVAECAEERYRLLEQRDKILRQARPK      675
H.sapiens   568 HQFEGDS-FDSASEGS---EGLGPCVLSLSTLIGTVAETSKEKYRLLDQRDRIMRQARVK      623
G.gallus    386 LQFEVDL-FDSSSEGQN-SEAVGASLSSLSNLVGLVAETSEERYRLLDQRDKIMRQARIK      443
                  *  :  ::. .:       :      *. *   : :  : .  ::::    : :   .

                               203                                238
S.cerevisae 187 AKDLYDAIVFQGTCLDMCPTFERSRRNVEYTVYSYEKNQPNDKKASRTKALKVFARPAAA      246
D.rerio     676 RTDLDMSKVFVGTCPDMCPEKERYMRETRNQLSVFEV-VPDTEKVDHYAAIKEYSRSSAD      734
H.sapiens   624 RTDLDKARTFVGTCLDMCPEKERYMRETRSQLSVFEV-VPGTDQVDHAAAVKEYSRSSAD      682
G.gallus    444 RTDLGKAKTIVGTCPDMCPEKERYMRETRNQLSIFEL-LLGSDKVNHAAAIKEYSRSSAD      502
                 .**  : .: *** ****  **  *:..  :  :*    . .:..:  *:* ::* :*

                        256                               286    293
S.cerevisae 247 AAPPLPSDVRPPHILVKTLDYIVDNLLTT----LPESEGFLWDRMRSIRQDFTYQNYSGP      302
D.rerio     735 QEEPLPHELRPLPVLSMTMDYLVTQIMDQGEGNCRDWYDFVWNRTRGIRKDITQQHLCDP      794
H.sapiens   683 QEEPLPHELRPLPVLSRTMDYLVTQIMDQKEGSLRDWYDFVWNRTRGIRKDITQQHLCDP      742
G.gallus    503 QEEPLPHELRPSEVLSMTMDYLVTNIMDQGEGNYREWYDFVWNRTRGIRKDITQQHLCNP      562
                   *** ::**  :*  *:**:* :::        :  .*:*:* *.**:*:* *: ..*
```

**Figure S2:** ClustalOmega alignment of the *S. cerevisiae* Sac3 sequence with vertebrate homologues indicates substantial sequence similarity in the region containing the extended loop (Sac3 residues 90-125) and the three additional helices (Sac3 residues 126-250), consistent with these structural features being conserved. Sac3 Arg256 (red), which is buried in the crystal structure, is conserved, together with the residues surrounding it (cyan) such as Met203 and Asp293, together with Lys238 and Ser289 that are linked to Arg256 through H-bonds to S213. The level of conservation is indicated below the sequences with * indicating identity, : indicating high homology, and . indicating lower homology. This alignment was obtained using the EBI server (http://www.ebi.ac.uk/Tools/msa/clustalo).
